# *Sargassum fusiforme* polysaccharide attenuates high‐sugar–induced lipid accumulation in HepG2 cells and *Drosophila melanogaster* larvae

**DOI:** 10.1002/fsn3.2521

**Published:** 2021-08-12

**Authors:** Dan He, Liping Yan, Jiaqi Zhang, Fang Li, Yu Wu, Laijin Su, Peichao Chen, Mingjiang Wu, Jong‐il Choi, Haibin Tong

**Affiliations:** ^1^ College of Life and Environmental Science Wenzhou University Wenzhou China; ^2^ Department of Biotechnology and Bioengineering Chonnam National University Gwangju South Korea

**Keywords:** abnormal development, high‐sugar diet, lipid‐lowering effect, *Sargassum fusiforme* polysaccharide

## Abstract

Lipid accumulation is a major factor in the development of non‐alcoholic fatty liver disease (NAFLD). Currently, there is a lack of intervention or therapeutic drugs against NAFLD. In this study, we investigated the ability of *Sargassum fusiforme* polysaccharide (SFPS) to reduce lipid accumulation induced by high sugar in HepG2 cells and *Drosophila melanogaster* larvae. The results indicated that SFPS significantly (*p* < .01) decreased the accumulation of lipid droplets in high sugar–induced HepG2 cells. Furthermore, SFPS also suppressed the expression of *Srebp* and *Fas* (genes involved in lipogenesis) and increased the expression of *PPARɑ* and *Cpt1* (genes that participated in fatty acid β‐oxidation) in these cells. SFPS markedly reduced the content of triglyceride of the third instar larvae developed from *D. melanogaster* eggs reared on the high‐sucrose diet. The expression of the *Srebp* and *Fas* genes in the larvae was also inhibited whereas the expression of two genes involved in the β‐oxidation of fatty acids, *Acox57D‐d* and *Fabp,* was increased in the larval fat body (a functional homolog of the human liver). We also found that SFPS ameliorated developmental abnormalities induced by the high‐sucrose diet. These results of this study suggest that SFPS could potentially be used as a therapeutic agent for the prevention and treatment of NAFLD.

## Graphical abstract

In the present study, we first demonstrated that polysaccharide (SFPS) from medicinal and edible brown alga *Sargassum fusiform* showed lipid‐lowering activity by inhibiting lipogenesis and promoting fatty acid β‐oxidation in vitro and in vivo. We also found that high sugar–induced developmental retardation was ameliorated by SFPS activity. Meanwhile, SFPS is a natural phytochemical that reduces hepatic lipid accumulation, suggesting that it has potential applications in the prevention or treatment of nonalcoholic fatty liver disease and health promotion.

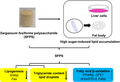

## 1 INTRODUCTION

Chronic consumption of sugar‐rich beverages or foods promotes the development of non‐alcoholic fatty liver disease (NAFLD) and reduces life expectancy (Tilman & Clark, 2014). Among the various sugars, sucrose is widely used as an inexpensive sweetener in beverages or foods; upon ingestion, sucrose is converted to glucose and fructose, which are subsequently absorbed and transported by the blood to the cells and tissues to be further metabolized. In humans, excessive sugar intake disrupts normal lipid metabolism and increases the de novo synthesis of fatty acids, thereby promoting their accumulation in the human liver as triacylglycerol (Bray, 2013; Manti et al., 2014). Notably, the preference for sweetness in humans develops early in life, to the extent that children and adolescents are prone to consuming large amounts of sugar‐rich beverages or foods, which can increase their risk of NAFLD (Codella et al., 2017; Jensen et al., 2018; Prinz, 2019). The pathogenesis of NAFLD has been attributed to a “two‐hit” theory, with the first hit being lipid accumulation in hepatocytes (Tessari et al., 2009), which is considered to be an early stage of NAFLD. It plays a key role in the progression of NAFLD. Therefore, the main strategy for the treatment and prevention of NAFLD is to reduce lipid accumulation.

*Drosophila* is a common model for studying human metabolic diseases because it not only has organs with similar functions to those of humans but also shares the same metabolic pathways with humans. For example, the *D*. *melanogaster* fat body is considered to be a functional homolog of the human liver (Musselman & Kühnlein, 2018; Ugur et al., 2016). In *Drosophila*, a high‐sucrose diet increases lipid accumulation in the fat body, which is prone to the development of NAFLD (Kim et al., 2021; Tian et al., 2011). Therefore, this model can be used to screen for compounds that can be used to prevent and treat NAFLD (Sanhueza et al., 2021).

Algae are widely consumed in Asia (Mišurcová et al., 2012). Several studies have shown that algal polysaccharides can prevent or treat metabolic diseases such as obesity and NAFLD (Chater et al., 2015; Heeba & Morsy, 2015). *Sargassum fusiforme*, which is rich in water‐soluble polysaccharide, is a commercially cultivated alga in China, Japan, and Korea (Chen et al., 2016). Previous studies have reported that *S. fusiforme* polysaccharide possesses antioxidant, antitumor, immune‐enhancing, anti‐aging, blood glucose–lowering, anticoagulation, antiviral, and antibacterial activities, with potential applications in the pharmaceutical and cosmeceutical industries (Zhang et al., 2020). However, there have been few studies focusing on the lipid‐lowering effect of SFPS. In this study, we investigated the lipid‐lowering effect of *S. fusiforme* polysaccharide (SFPS) in high sugar–induced HepG2 cells and *D. melanogaster* larval model. The results of our analysis would help expand the scope of the use and application of SFPS.

## 2 MATERIALS AND METHODS

### 2.1 Materials and chemicals

*Drosophila melanogaster w* ^1118^ was provided by Qidong Fungene Biotechnology (Jiangsu, China). Assay kits for glucose, triglyceride, and total protein were provided by Shenzhen Icubio Biomedical Technology Co., Ltd (Shenzhen, China). TRIzol reagent was purchased from Invitrogen (Carlsbad, CA, USA). PrimeScript^TM^ RT Master Mix (Perfect Real Time) was obtained from Takara Biomedical Technology Co., Ltd. (Dalian, China). TransStart Top Green qPCR SuperMix was purchased from TransGen Biotech (Beijing, China). Minimum essential medium (MEM), fetal bovine serum (FBS), MEM nonessential amino acids (100×), L‐glutamine (100×), and sodium pyruvate were provided by Gibco (Grand Island, NY, USA). Penicillin–streptomycin solution (100×) was obtained from Biosharp Life Sciences (Anhui, China). All other chemical reagents used in this study were of analytical grade.

### 2.2 Extraction and characterization of *S. fusiforme* polysaccharide

**FIGURE 1:**
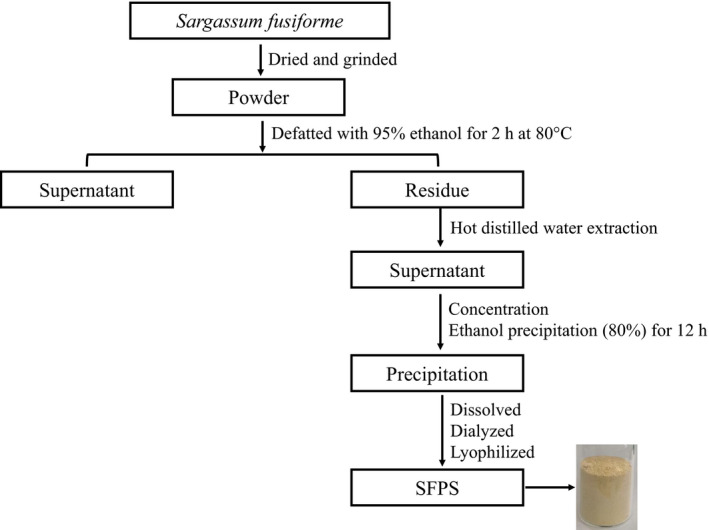
Diagram outlining the steps involved in the extraction of *S. fusiforme* polysaccharide (SFPS)

The *S. fusiforme* polysaccharide SFPS was prepared as previously reported (Chen et al., 2016) as shown in Figure 1. The sulfate content, protein content, uronic acid, molecular weight, and monosaccharide composition of SFPS were analyzed by the BaCl_2_–gelatin method, Bradford method, *m*‐hydroxydiphenyl method, and 1‐phenyl‐3‐methyl‐5‐pyrazolone (PMP) pre‐column derivatization and HPLC method (Chen et al., 2016; Wu et al.,^2019^). The yield of SFPS was 19.7%. It contained 6.9% sulfated group, 0.3% protein, and 90.9% sugar that included with 46.2% uronic acid, and the average molecular weight of SFPS was 80 kDa. The detailed composition of the monosaccharides in SFPS is glucose, glucuronic acid, xylose, rhamnose, mannose, mannuronic acid, galactose, fucose, and guluronic acid with the molar ratio of 1:3:4:5:4:9:7:28:39.

### 2.3 In vitro experiments

#### 2.3.1 Cell culture

HepG2 (human hepatocellular liver carcinoma) cells were purchased from the Cell Bank of Shanghai Institute of Biochemistry and Cell Biology. The cells were cultured in a MEM complete medium supplemented with 10% FBS and 1% penicillin–streptomycin at 37°C in a 5% CO_2_ incubator.

#### 2.3.2 Cell viability analysis

The viability of the cells treated with glucose was determined using the MTT assay. Briefly, HepG2 cells were treated with different concentrations of glucose (12.5, 25, 50, 100 mM) for 48 hr, and the cell viability was determined by an MTT analysis as described previously (Chen et al., 2016).

#### 2.3.3 Oil red O staining

HepG2 cells were seeded in a 24‐well plate for overnight and then cultured in the complete medium (control group), complete medium containing 50 mM glucose (glucose group), or 50 mM glucose plus 100 µg/ml SFPS (SFPS group) for 48 hr. After that, the cells were stained using an oil red O stain kit (for cultured cells) (Solarbio, G1262) according to the manufacturer's instruction. Images of the stained cells of each group were obtained using an inverted microscope (LEICA DMi8, Wetzlar, Germany). Oil red O–stained area was measured using Image J software. The concentration of SFPS was found to have no toxic effect on liver cells at a concentration of 100–1,000 μg/ml (Chen et al., 2016).

### 2.4 In vivo experiments

#### 2.4.1 Preparation of media

Three types of diets were prepared for the experiments that examined the lipid‐lowering effect of SFPS on *Drosophila melanogaster*. A standard diet containing 3% sucrose was prepared by dissolving 20 g corn, 8 g sucrose (3%) or 87.5 g (35%), 16 g glucose, 8 g yeast, 0.18 g calcium chloride, 1.75 g agar, and 2 ml propionic acid in hot water in a total volume of 250 ml. The diet was then allowed to solidify. To prepare the high‐sucrose diet which contained 35% sucrose, the same preparation was carried out except that the amount of sucrose in the diet was increased to 87.5 g of sucrose. As for the high sucrose plus SFPS diet, 6.25 g of SFPS was added to the preparation in addition to 87.5 g of sucrose. The final concentration of SFPS in the diet was 25 mg/ml.

#### 2.4.2 *Drosophila melanogaster* culture and treatment

*Drosophila melanogaster* was reared on a standard diet in a 25℃ incubator with a relative humidity of 60%–70% and under a 12 hr light/dark cycle. The embryos were collected on grape juice agar plates with yeast extract (1%, *m*/*v*) and then randomly separated into three groups as follows:

1. Control group: *D*. *melanogaster* embryos (150 eggs) were reared on a standard diet (8 ml).
2. SD group: *D*. *melanogaster* embryos (150 eggs) were reared on the high‐sucrose diet (8 ml).
3. SFPS group: *D*. *melanogaster* embryos (150 eggs) were reared on the high sucrose +SFPS diet (8 ml).

#### 2.4.3 Phenotypic analysis

*Drosophila melanogaster* larval development was monitored to track the changes from the larval to adult stages. The body weight was recorded for three replicates of 10 larvae/tube and 10 pupae/tube. Pupariation rates were recorded every 24 hr and analyzed using Equation (1) below. *Drosophila* larvae, pupae, and adults were photographed using a stereomicroscope (Nikon SMZ1270, Japan). Additionally, adult wings were photographed using an inverted biological microscope (Leica DMi1, Germany). The length and area of the adult wing were analyzed using ImageJ software (National Institutes of Health, Bethesda, MD, USA). Pupal volume was calculated using Equation (2) (Parisi et al., 2013).

(1) Pupariationrate(%)=[numberofpupae/totalnumberofembryos]×100%

(2) $$\text{Pupal}\;\text{volume}\;({\text{mm}}^{3})=(4\pi /3)\times (L/2)\times (l/2){\;}^{2}$$

where is “L” is the pupal length and “l” is the pupal width.

#### 2.4.4 Biochemical analysis

The control, SD, and SFPS groups of *D. melanogaster* larvae (*n* = 10) were separately collected in 1.5 ml microcentrifuge tubes, ground in 0.1% PBST (PBS containing 0.1% Triton X‐100), and centrifuged at 10,000 × *g* at 4℃ for 10 min. The supernatant from each sample was collected and separated into two tubes. One of the tubes was used to measure the total protein content using an automatic biochemical analyzer (Shenzhen icubio Biomedical Technology Co., Ltd.) equipped with a total protein assay kit. The other tube was heated at 70°C for 5 min to deactivate the endogenous enzymes, and the debris was then removed by centrifugation and the triglyceride and glucose contents in the clear supernatant were analyzed using a commercial assay kit.

### 2.5 Quantitative polymerase chain reaction (qPCR) analysis

Total RNA was extracted from the larvae, larval fat body, and HepG2 cells using the TRIzol reagent. First‐strand cDNA was synthesized from the RNA using a PrimeScript^TM^ RT Master Mix according to the manufacturer's instruction. The cDNA was then analyzed by qPCR using gene‐specific primers (Table 1). Quantitative PCR was performed in a LightCycler 96 (Roche, Switzerland) using TransStart Top Green qPCR SuperMix. The relative quantitation of the target‐gene mRNA level was performed using the 2^−ΔΔCt^ method.

**TABLE 1:** Sequences of gene‐specific primers used in qPCR analysis

Organism	Gene name	Primer sequence (5′–3′)
*Homo sapiens*	*Fas*	GGACAGAGCAACTACGGCTT
		GTGTCGTTGGTGCTCATCGT
	*Srebp*	GTGCTCTGCGAGTGGATG
		CAGGTTGGTGGCAGTGAG
	*Cpt1*	GTTACGACAGGTGGTTTGAC
		CGTTTGCCAGAAGATTTGCG
	*PPARɑ*	CATCCCAGGCTTCGCAAACT
		TCCATACGCTACCAGCATCC
	*β‐actin*	TGGATCAGCAAGCAGGAGTA
		TCGGCCACATTGTGAACTTT
*Drosophila melangaster*	*Acox57D‐d*	CCACGAGATACTCGGCAGTG CCGTAGCGATCTCAGGGAAG
	*Fabp*	TGTAGACGCGCACGCACTTA
		AGCATCATCACCCTGGATGG
	*Srebp*	GGCAGTTTGTCGCCTGATG
		CAGACTCCTGTCCAAGAGCTGTT
	*Fas*	CAACAAGCCGAACCCAGATCTT
		CAAAGGAGTTCAGGCCGATGAT
	*Dilp2*	AGCAAGCCTTTGTCCTTCATCTC
		ACACCATACTCAGCACCTCGTTG
	*Dilp3*	AAGCTCTGTGTGTATGGCTT
		AGCACAATATCTCAGCACCT
	*FoxO*	CGAGAGTCCGCTCCACAG
		AAGATCCTGCGCCCTAATG
	*Thor*	CCATGATCACCAGGAAGGTTGTCA
		AGCCCGCTCGTAGATAAGTTTGGT
	*RpL32*	GACAGTATCTGATGCCCAACA
		CTTCTTGGAGGAGACGCCGT

### 2.6 Statistical analysis

Statistical analysis was performed using GraphPad Prism version 8.00 (GraphPad, San Diego, CA, USA). One‐way and two‐way ANOVA followed by Tukey's multiple comparison test was performed to determine the significant differences among groups. Statistical significance was considered at the *p* <.05 level.

## 3 RESULTS

### 3.1 Effect of glucose on the viability of HepG2 cells

**FIGURE 2:**
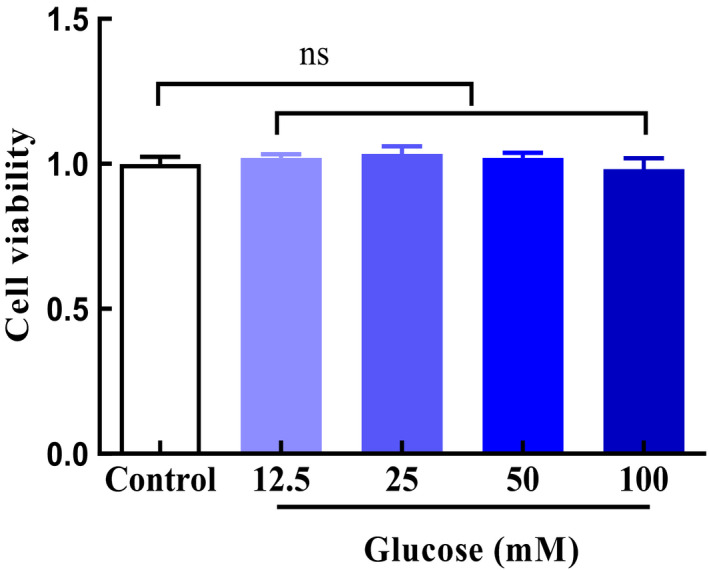
Effect of glucose on HepG2 cells viability. HepG2 cells were incubated with glucose (12.5, 25, 50, 100 mM) for 48 hr, and the cell viability was then measured with the MTT assays. Data are presented as the means ±standard deviation from three independent experiments. ns: no significant difference

The effect of glucose on HepG2 cells was determined by measuring the viability of the cells following treatment with different concentrations of glucose. No significant difference in the cell viability was detected between untreated (control) cells and glucose‐treated cells for glucose concentration up to 50 mM compared with the untreated (control) cells. However, at 100 mM glucose, there was a slight reduction in cell viability (Figure 2), suggesting that the range of glucose concentration tested exerted little or no toxicity on HepG2 cells. Therefore, 50 mM glucose was subsequently used to induce lipogenesis in HepG2 cells.

### 3.2 Effect of SFPS on high‐glucose‐induced lipid accumulation in HepG2 cells

**FIGURE 3:**
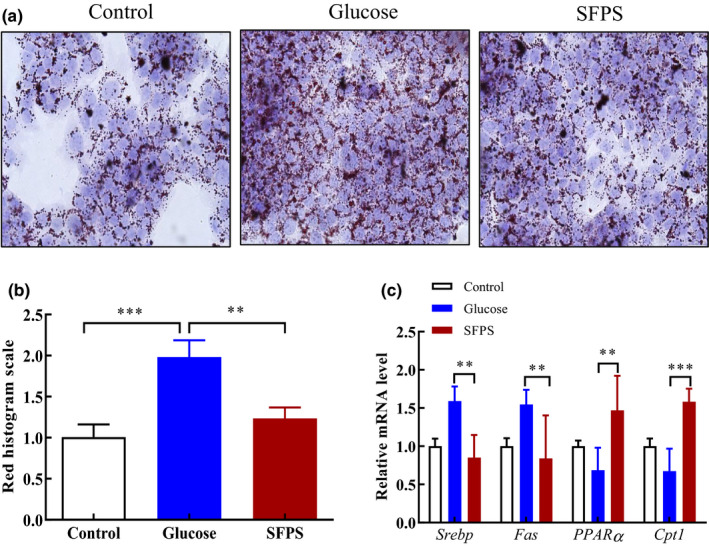
Effect of *S. fusiforme* polysaccharide (SFPS) on high glucose–induced lipid accumulation in HepG2 cells. HepG2 cells were treated with 50 mmol/L glucose or 50 mmol/L glucose +100 µg/ml SFPS for 48 hr and then subjected to different analyses. (a) Oil red O staining. (b) Plot showing the quantitation of lipid as identified by oil red O staining shown in (a). (c) Quantitative analysis of the transcript levels of gene associated with lipogenesis and fatty acid β‐oxidation. Data are the means ±standard deviation from three independent experiments. **p <.01, ***p <.001

The effect of SFPS on lipid accumulation in glucose‐treated HepG2 cells was determined by oil red O staining (ORO), a method that is often used to detect neutral lipids and the content of lipid droplets (Mehlem et al., 2013). HepG2 cells induced with glucose in the presence of SFPS exhibited decreased lipid accumulation compared with cells that were induced with glucose without SFPS (Figure 3a and b). Subsequent qPCR analysis of lipid metabolism‐related genes in these cells revealed reduced transcript levels of the *Srebp* and *Fas* genes (Figure 3c). Furthermore, the transcript levels of *Cpt1* and *PPARɑ* in these cells also increased by 2.3‐ and 2.1‐fold, respectively, suggesting that SFPS could promote β‐oxidation of fatty acids and inhibit lipogenesis.

### 3.3 Effects of SFPS on the lipid accumulation of high sucrose–fed *D. melanogaster* larvae

**FIGURE 4:**
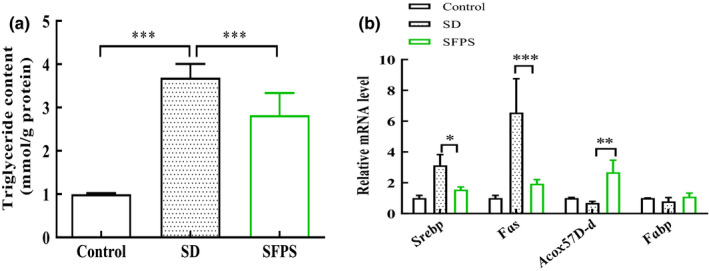
Effect of SFPS on lipid accumulation in *Drosophila melanogaster* larval fat body. (a) Triglyceride content in the third instar larvae. (b) Transcript levels of genes involved in lipogenesis and β‐oxidation of fatty acids in the third instar larval fat body as determined by qPCR. Data are presented as means ±standard deviation from three independent experiments. *p <.05, **p <.01, ***p <.001

To further determine that SFPS also has a lipid‐lowering effect in vivo, we analyzed the triglyceride content of *D*. *melanogaster* larvae reared on a high‐sucrose diet since triglyceride is the major form of stored fat. Third instar larvae reared on the high‐sucrose diet showed an increase in the triglyceride content compared with the control larvae (Figure 4a). In contrast, third instar larvae that were reared on the high‐sucrose diet plus SFPS medium (SFPS group) showed a significantly reduced content of triglyceride. To determine the potential mechanisms by which SFPS reduced lipid accumulation in the high sucrose–fed larvae, several lipogenesis genes in the fat body of these larvae were examined using qPCR. Fatty acid synthetase (FAS) is a lipogenic factor activated by the transcription factor SREBP. The mRNA level of *Fas* and *Srebp* in the SFPS group decreased by 0.3‐ and 0.5‐fold, respectively, relative to the SD group, and the reduction was significant for both genes (Figure 4b). This indicated that SFPS could prevent an increase in lipogenesis in *D. melanogaster* larvae reared on the high‐sucrose diet. Further analysis of lipid catabolism–related genes revealed a reduction in the transcript levels of *Acox57D‐d* (acyl‐Coenzyme A oxidase at 57D distal) and *Fabp* (fatty acid‐binding protein) in the SD group, but the transcript levels of both genes were significantly upregulated in the SFPS group. These results suggest that the lipid‐lowering effect of SFPS might occur through the regulation of lipid metabolism–related genes, consequently leading to reduced lipogenesis and increased fatty acid β‐oxidation in the larval fat body.

### 3.4 Effects of SFPS on the *D. melanogaster* larval development

**FIGURE 5:**
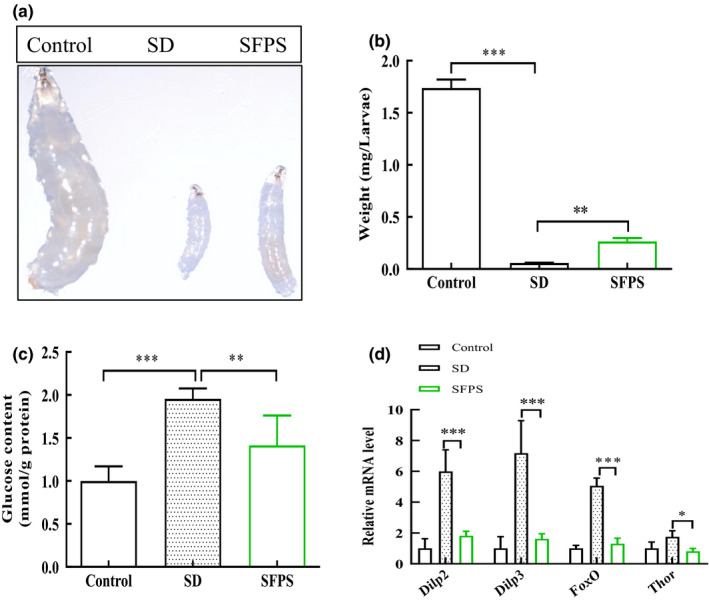
Effect of SFPS on larval development in for *Drosophila melanogaster* larvae fed with a high‐sucrose diet. The eggs produced by mating between female and male *D. melanogaster* individuals were reared on standard diet (control), high‐sucrose diet (SD), and high‐sucrose plus SFPS diet (SFPS) for 120 hr and then various parameters of growth, glucose content, and insulin‐related gene were analyzed. (a) Representative images showing the larvae reared on the three different treatments. (b) Weight. (c) Glucose content. (d) Insulin signal–related gene expression. Data are the means ±standard deviations from three independent experiments. *p <.05, **p <.01, ***p <.001

*Drosophila* development was monitored by measuring the growth defects by comparing larval phenotypes at 120 hr, a time at which synchronized cultures of normal larvae would normally reach the wandering third instar stage with the commencement of puparium formation (Rulifson et al., 2002). A previous study has shown that larval development can be assessed by morphometric and weight measurements (Ugur et al., 2016). The larval size of the SD group was reduced compared with the larval size of the control group, and the larval size of the SFPS group was larger than that of the SD group (Figure 5a). The larval weight of the SFPS group was also significantly increased relative to that of the SD group, with the increase being about 4.6‐fold that of the SD group (Figure 5b). It has been shown that excessive fat accumulation can cause lipotoxicity, which plays a role in insulin resistance and islet β‐cell dysfunction (Yazıcı & Sezer, 2017). Thus, the effect of SFPS on the expression of the genes coding for insulin‐like peptide 2 (*Dilp2*) and insulin‐like peptide (*Dilp3*) was also investigated. Significantly higher levels of *Dilp2* and *Dilp3* mRNA were found in the SD group than in the control group, with the increase amounted to 6.0‐ and 7.2‐fold for *Dilp2* and *Dip3*, respectively, (Figure 5d). However, these larvae showed an increased level of glucose (Figure 5c), a physiological state similar to insulin resistance. In *Drosophila*, FOXO (Forkhead transcription factor) is an important mediator of insulin signaling, and it is activated to induce insulin resistance and inhibits growth through the action of target genes such as *Thor* (Jünger et al., 2003; Luong et al., 2006). The transcript levels of *FoxO* and *Thor* in the SD group larvae also increased relative to the control group (5.0‐fold for *FoxO* and 1.8‐fold for *Thor*). The data were consistent with the phenotypic abnormalities and metabolic disorders reported by other investigators for larvae reared under a high‐sucrose diet. Thus, SFPS could reduce developmental abnormalities in *D. melanogaster* larvae by reducing lipid accumulation.

### 3.5 Effects of SFPS on *D. melanogaster* pupal development

**FIGURE 6:**
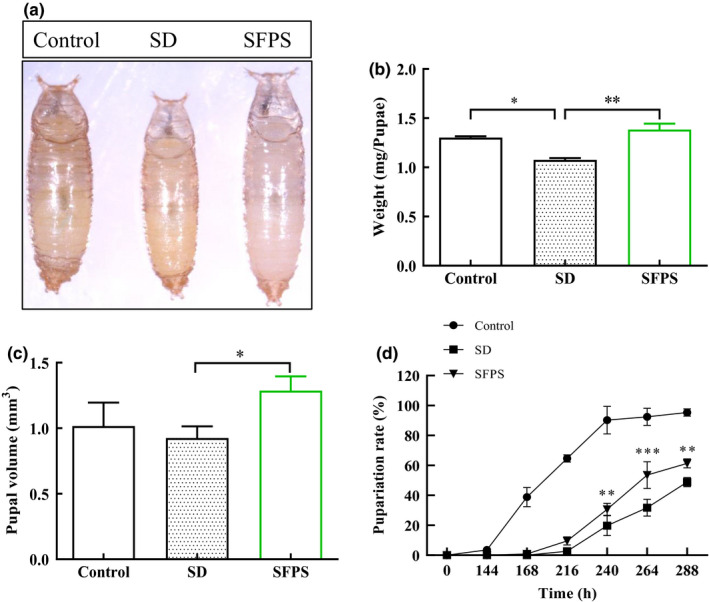
Effect of SFPS on *Drosophila melanogaster* pupal development. The larvae were fed a normal diet (control group), high‐sucrose diet (SD group) or high‐sucrose diet +SFPS (SFPS group) until they reached the pupal stage. The pupariation rate, pupal weight, and volume were then determined. (a) Representative image showing the pupae from the three different treatments. (b) Pupariation rate. (c) Pupal weight. (d) Pupal volume. Data are the means ±standard deviations from three independent experiments. *p <.05, **p <.01, ***p <.001

The effect of SFPS on *D. melanogaster* pupal development was investigated. As shown in Figure 6a, the pupal size of the SD group was obviously smaller than that of the control group, whereas both the control and SFPS groups had similar pupal sizes. The pupal weight and volume of the SD group showed a significant decrease relative to the control group, but the pupal weight and volume of the SFPS group were similar to those of the control group (Figure 6b and c). This clearly demonstrated that SFPS could prevent the loss of pupal weight and volume caused by the high‐sucrose diet. Among the three groups, the SD group also had the lowest pupariation rate, whereas the control group had the highest pupariation rate (Figure 6d). The SFPS group had a slightly higher pupariation rate than the SD group, but the difference was significant at 240 hr and beyond. The results demonstrated that SFPS feeding could suppress the high sucrose–induced delay in larval body development and attenuated the abnormal growth exhibited by *Drosophila* from the larval stage to the pupal stage.

### 3.6 Effects of SFPS on the *D. melanogaster* adult development

**FIGURE 7:**
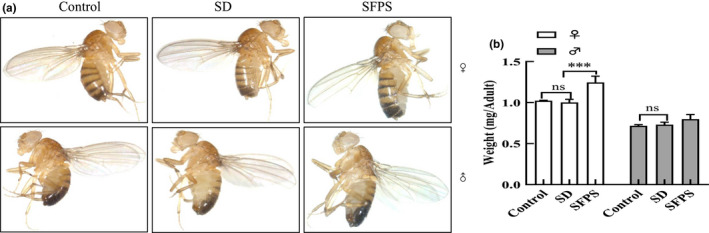
Effect of SFPS on *Drosophila melanogaster* adult development. The larvae were fed a normal diet (control group), high‐sucrose diet (SD group) or high‐sucrose diet +SFPS (SFPS group) until they reached the adult stage, and the body weights of the flies were then measured. (a) Representative image showing the adult from the three different treatments. (b) Body weight. Data are presented as means ±standard deviation from three independent experiments. ns: no significant difference, ***p <.001

**FIGURE 8:**
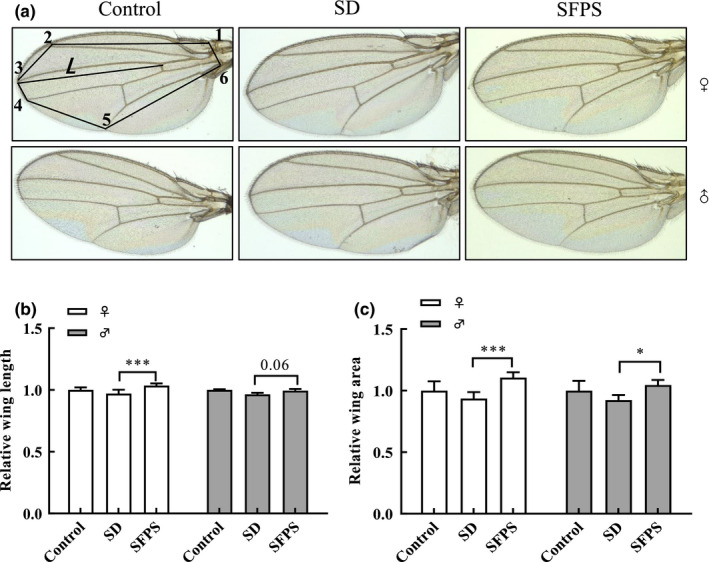
Effect of SFPS on *Drosophila melanogaster* wing development. The wings of female and male adults from the control, SD, and SFPS groups were analyzed with respect to wing length and area. (a) Representative image showing the adult wing from flies from the three different groups. (b) Relative wing length, as determined from the length of the line segment *L* in (a). (c) Wing area. Wing area was calculated from the area enclosed by the six points connected by the black line segments (1–6) in (a). Data are the means ±standard deviation from three independent experiments. *p <.05, ***p <.001

The effect of SFPS on *D. melanogaster* body size and weight was also evaluated for the adult individual fed with the high‐sucrose diet. No significant difference was observed in body size and weight between females and males between the SD and control groups (Figure 7a and b), while both males and females in the SFPS group appeared to have a larger body size and weight, though only the females displayed a significant increase in body weight over the SD group. Wing area and wing length were also measured to provide additional indicators of adult development (Hariharan & Serras, 2017). As shown in Figure 8a, Line *L,* which extends from the anterior crossvein to the end of the second longitudinal vein, was used to measure the wing length, while the black line segment superimposed on the wing connects the six points (1–6) used to determine the area of the wing. Adult flies (both males and females) of the SFPS group possessed longer and larger wings than those in the SD group (Figure 8b and c). Thus, SFPS could correct the negative impact of high sucrose and ensure the proper development of the larvae to the adult stage.

## 4 DISCUSSION

Regular consumption of sugar‐sweetened beverages has been linked to an increased risk of metabolic diseases in children and adolescents, prompting a worldwide health concern (Bradwisch et al., 2020; Flieh et al., 2020; Schwimmer et al., 2019). Among the metabolic diseases that have attracted much attention is NAFLD. At present, no drug has been approved by the FDA to treat NAFLD in children (Friesen et al., 2021). Several studies have demonstrated the potential of algae‐derived active ingredients such as alginate and xanthigen in the prevention of NAFLD (Abidov et al., 2010; Kawauchi et al., 2019). Therefore, we speculated that polysaccharide obtained from *S. fusiforme* may also have therapeutic potential against NAFLD.

High‐sugar diet can alter the metabolism of fatty acids in the liver, resulting in the accumulation of lipid within the liver cells (Alves‐Bezerra & Cohen, 2017). HepG2 cells display many genotypic features of normal human hepatocytes and are widely used to screen for active compounds via in vitro models (Donato et al., 2015). Using HepG2 cells to represent liver cells showed that SFPS could significantly inhibit glucose‐induced lipid accumulation in the cells exposed to a high concentration of glucose in the medium. Many studies have reported that active compounds with lipid‐lowering effect can prevent the accumulation of lipid in HepG2 cells by inhibiting the expression of *Srebp* and *Fas* (Do et al., 2013; Kang & Koppula, 2014; Pil Hwang et al., 2013). FAS and SREBP are important enzymes in liver lipogenesis (Lüersen et al., 2019). The lipid‐lowering effect of SFPS was also linked to its inhibition of high glucose‐induced *Fas* and *Srebp* expression (Figure 3). In addition, the activation of *PPARα* and *Cpt1*, which also participate in the β‐oxidation of fatty acids, and their activation can lead to a decreased level of lipid (Zhang et al., 2019). Therefore, SFPS might exhibit a lipid‐lowering effect via inhibiting lipogenesis and increasing the breakdown of fatty acid via β‐oxidation in our in vitro model. The underlying mechanism by which SFPS elicited its lipid‐lowering effect is consistent with that demonstrated for ginseng seed oil, which can promote fatty acid breakdown via β‐oxidation in HepG2 and rat hepatocytes (Kim et al., 2018).

To explore whether the lipid‐lowering effect of SFPS observed in the cell system might also occur in vivo, the effect of SFPS on *D. melanogaster* larvae fed with a high‐sugar diet was investigated. Although *D. melanogaster* has no liver, it does have a fat body, which is a liver analog, where fat can accumulate from excess glucose in the diet. Furthermore, the pathological accumulation of lipids in the fat body induced by high sugar can also lead to a shortened lifespan for the fly (Musselman et al., 2011). SFPS was found to reduce the level of triglyceride in *D. melanogaster* larvae fed with a high‐sugar diet, resulting in less accumulation of lipid in the fat body. The reduction in lipid accumulation was linked to an increase in *β*‐oxidation of fatty acids, mediated by increased expression of *Acox57D‐d* and *Fabp* in the fat body (Figure 4). This may represent an important mechanism by which SFPS exerts its lipid‐lowering effect in *D. melanogaster* larvae. Lipid accumulation in hepatocytes leads to hepatic steatosis, which is an early feature of NAFLD (Tailleux et al., 2012). We speculated that SFPS might prevent the progression of NAFLD by reducing lipid accumulation. Excessive fat accumulation plays a role in insulin resistance and islet β cell dysfunction, which in turn disrupts glucose metabolism in the body (Yazıcı & Sezer, 2017). Interestingly, SFPS, a seaweed polysaccharide, could improve insulin signaling, thereby alleviating the extent of abnormal development caused by defects in insulin signaling. Protection by SFPS against tissue damage caused by high sugar was obvious at the larval stage, and this might constitute an important factor by which SFPS rescued the developmental defects inflicted by high sugar in the pupal and adult stages. Metabolic imbalance not only can trigger a developmental delay of the body and organs but can also decrease the survival rate of the pupa (Murphy et al., 2006). The pupariation rate was significantly reduced by the high‐sucrose diet, but a significant increase in pupariation rate was observed for the pupae fed with the high‐sucrose diet containing SFPS (Figure 6). Judging from the lipid‐lowering effect of SFPS, both in HepG2 cells and *Drosophila*, SFPS might be a suitable agent for the prevention or treatment of obesity or non‐alcoholic fatty liver disease in developing children and adolescents.

In this study, SFPS, a sulfated polysaccharide extracted from *S. fusiforme* was shown to suppress lipid accumulation by increasing fatty acid breakdown via β‐oxidation and reduced lipid synthesis. Using *D*. *melanogaster* larvae raised on the high‐sucrose diet, the lipid‐lowering activity of SFPS was also manifested in the correction of developmental abnormalities adult, enabling larvae which otherwise suffered from developmental abnormalities to develop into normal adult flies. The findings of this study seem to suggest that polysaccharide derived from *S. fusiforme* might be developed into a functional food for the intervention in NAFLD and promotion of health.

## CONFLICT OF INTEREST

The authors declare no conflict of interests.

## ETHICAL APPROVAL

Not applicable.

## Data Availability

The authors confirm that the data supporting the findings of this study are available within the article.
